# The changes of the calf-vein deformation and femoral vein peak velocity during ankle pump exercise with or without graduated compression stockings

**DOI:** 10.1186/s12891-022-05400-y

**Published:** 2022-05-10

**Authors:** Zaikai Zhuang, Dongmei Ai, Yao Yao, Liming Zheng, Jianghui Qin, Dongyang Chen, Senlin Chai, Jun Lu, Qing Jiang, Xinhua Li

**Affiliations:** 1 State Key Laboratory of Pharmaceutical Biotechnology, Division of Sports Medicine and Adult Reconstructive Surgery, Department of Orthopedic Surgery, Nanjing Drum Tower Hospital Clinical College of Nanjing Medical University, 321 Zhongshan Road, Nanjing, 210008 Jiangsu People’s Republic of China; 2 Branch of National Clinical Research Center for Orthopedics, Sports Medicine and Rehabilitation, Nanjing, Jiangsu People’s Republic of China; 3 Department of Rehabilitation Medicine, Nanjing Drum Tower Hospital, The Affiliated Hospital of Nanjing University Medical School, 321 Zhongshan Road, Nanjing, 210008 Jiangsu People’s Republic of China

**Keywords:** Ankle pump exercise, Graduated compression stockings, Vein cross-sectional area, Venous blood velocity, Deep venous thrombosis, Thromboprophylaxis

## Abstract

**Objectives:**

To analyze the changes of lower limb hemodynamics parameters before and after wearing graduated compression stockings (GCS) during ankle pump exercise in patients preparing for arthroplastic surgery.

**Method:**

The leg veins of 16 patients awaiting arthroplasty were analyzed using a Sonosite M-Turbo ultrasound system during ankle pump exercise with or without GCS. The age of them was 70 ± 7 years (mean ± SD) (range 56—82 years) and body mass index was 25.8 ± 3.0 kg/m2 (range 18.0—30.5 kg/m2). Measured data including the cross-sectional area (CSA), anteroposterior (AP) diameter and lateromedial (LM) diameter of the soleus vein (SV), posterior tibial vein (PTV) and great saphenous vein (GSV). Additionally, the peak velocities of femoral vein (FV) were also measured.

**Results:**

GCS could significantly decrease the cross-sectional area of SV, PTV and GSV in supine position at rest and maximum ankle plantar flexion. But the compression effect of GCS to SV and GSV was not observed during maximum ankle dorsiflexion. It was found that GCS application reduced the peak flow velocity of the femoral vein from 61.85 cm/s (95% CI = 50.94–72.75 cm/s) to 38.01 cm/s (95% CI = 28.42–47.59 cm/s) (*P* < 0.001) during ankle plantar flexion and decreased the femoral vein in these patients from 80.65 cm/s (95% CI = 70.37–90.92 cm/s) to 51.15 cm/s (95% CI = 42.58–59.73 cm/s) (*P* < 0.001) during ankle dorsiflexion. But this effect was not significant in supine position at rest.

**Conclusions:**

GCS could significantly reduce the peak flow velocity of the femoral vein during ankle pump exercise in the patients preparing for arthroplastic surgery.

## Introduction

The incidence of deep vein thrombosis in patients following joint replacement surgery without thromboprophylaxis is approximately 40%-60% [1]. And the rate of postoperative venous thromboembolism is about 1% despite pharmacological thromboprophylaxis [2]. Deep vein thrombosis (DVT), with common complications including post-thrombotic syndrome [3, 4] and pulmonary embolism [5, 6], not only brings a huge health burden but also generates a high economic burden to patients, families and society [7–9]. So, the early intervention of DVT has great clinical significance for patient after joint replacement surgery. Deep venous thrombosis prevention included mechanical prophylaxis, pharmacological prophylaxis and a combination of these [10]. Mechanical prophylaxis is considered to be one of the most necessary measures for the prevention of DVT and is the necessary complement to pharmacological prophylaxis. Moreover, mechanical prophylaxis can serve as an alternative to pharmacological prophylaxis in patients with coagulation disorders or at high risk of bleeding [11].

Graduated compression stockings are the most common method for mechanical thromboprophylaxis and effective rehabilitation treatment [12–14], which may increase the venous blood flow velocity and reduce venous diameters [15–17]. However, in clinical application, it is still controversial whether it can reduce the incidence of thrombosis after joint replacement surgery [18–20]. We conducted a study that focused on patients awaiting total knee arthroplasty in a supine position [21]. It was found that knee-length GCS could significantly compress the distal (calf) veins, but it had no significant changes in blood velocity of the proximal vein (including popliteal vein and femoral vein). These results may indicate that GCS are not effective in improving blood flow velocity while at rest in the supine position. It was guessed that the results we obtained might not be the same at rest and during exercise. There are relatively few such studies at present, and thus it is necessary to conduct further research.

Ankle pump exercise is now recognized as an effective prevention method of thromboprophylaxis for patients with early postoperative status and long-term bedridden status. A large portion of the patients were at a bed-bound status for various reasons in the early postoperative period, so postoperative early physical exercise in bed may have important preventive implications for the DVT [22, 23]. Previous studies have found that ankle pump exercise can improve both venous blood flow velocity and venous back flow [24, 25], which can decrease the incidence of postoperative DVT [26]. It has been demonstrated that combined ankle pump exercise with other mechanical device played a positive role in improving blood flow velocity [27], but the combination of ankle pump and GCS remains to be further studied. And despite the widespread use of ankle pump exercise and GCS in clinical, it is still unclear whether GCS should be used simultaneously with ankle pump exercise. Consequently, we would like to explore the effect of GCS on lower extremity venous blood flow velocity and venous deformation during ankle pump exercise.

The aim of this study was to analyze the changes of lower limb hemodynamics parameters without and with wearing GCS during ankle pump exercise in patients preparing for arthroplastic surgery. We hypothesized that GCS application significantly increased the peak flow velocity of the femoral vein and reduced the cross-sectional area of the calf-vein during ankle exercise in the patients before joint replacement. This study will help offer a theoretical basis for the reasonable mechanical prevention methods in patients after joint replacement surgery.

## Methods

### Participants

A total of 16 patients awaiting arthroplasty were analyzed. The age of them was 70 ± 7 years (mean ± SD) (range 56—82 years) and body mass index was 25.8 ± 3.0 kg/m2 (range 18.0—30.5 kg/m2). Exclusion criteria were as follows: surgery history involving the lower limbs, lower limb skin abnormalities, lower extremity neuropathy, lower extremity vascular lesions, deep vein thrombosis, varicosities. All methods were performed in agreement with the Declaration of Helsinki. Informed consent was obtained from all participants, and the present study was approved by the hospital Ethic Committee.

### Experimental design

The experimental started with a 15-min rest period in the supine position to allow hemodynamic stabilization. The first measurement was obtained while supine at rest before wearing GCS. Measured data including the cross-sectional area, anteroposterior diameter and lateromedial diameter of the soleus vein, posterior tibial vein and great saphenous vein. Additionally, the peak velocities of femoral vein were also measured. The specific measurement methods were performed as previously described [20]. The ankle plantar flexion training of the foot at an average rate of 15 flexions/min was performed 5 min rest after the first measurement. Five minutes later, the second measurement was obtained. Similarly, the ankle dorsiflexion training of the foot at an average rate of 15 flexions/min was performed 5 min rest after the second measurement and the third measurement was obtained after five minutes.

**Fig. 1:**
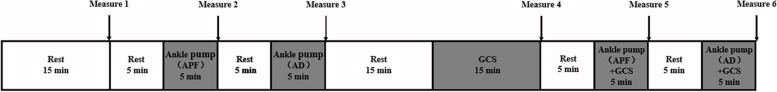
The schematic diagram of the experimental protocol

**Fig. 2:**
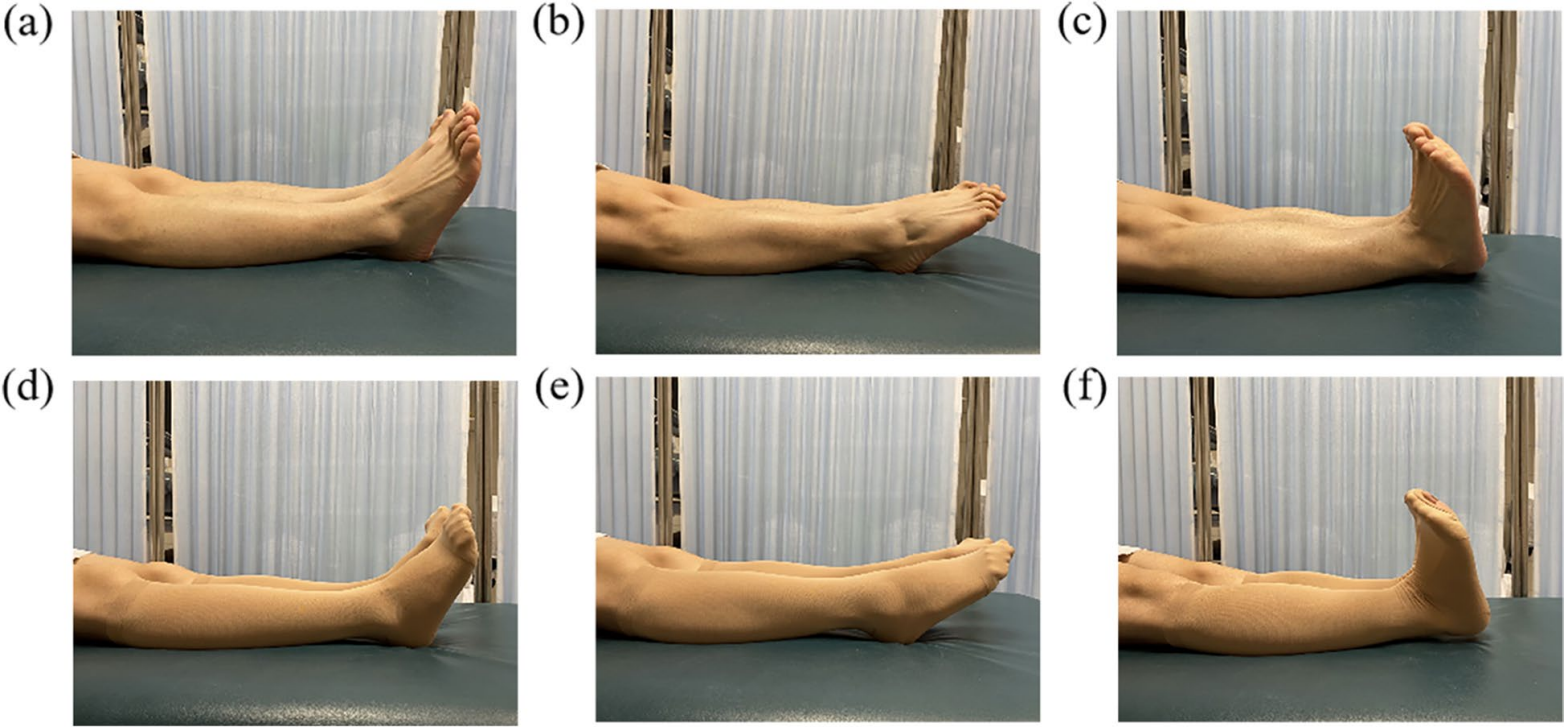
Six different states: **a** Rest, **b** Ankle plantar flexion, **c** Ankle dorsiflexion, **d** GCS alone, **e** combined GCS and Ankle plantar flexion, and **f** combined GCS and Ankle dorsiflexion

After this, the sequence of measurements, at rest, during ankle plantar flexion training and ankle dorsiflexion training, was repeated while wearing the GCS in the supine position (Fig. 1 and Fig. 2).

### Graduated compression stockings (GCS)

New knee-length gradient elastic compression stockings (Medical Supplies Pty Ltd, Haoshide, China) were used in each participant. According to the requirements of the instructions, the circumference measurements were made on the level of 10 cm underneath the inferior margin of patella and the ankle. The most suitable stocking was chosen based on these measurements. The pressure pattern in these stockings is 16–22 mmHg (compression class I).

### Ultrasonic measurements

**Fig. 3:**
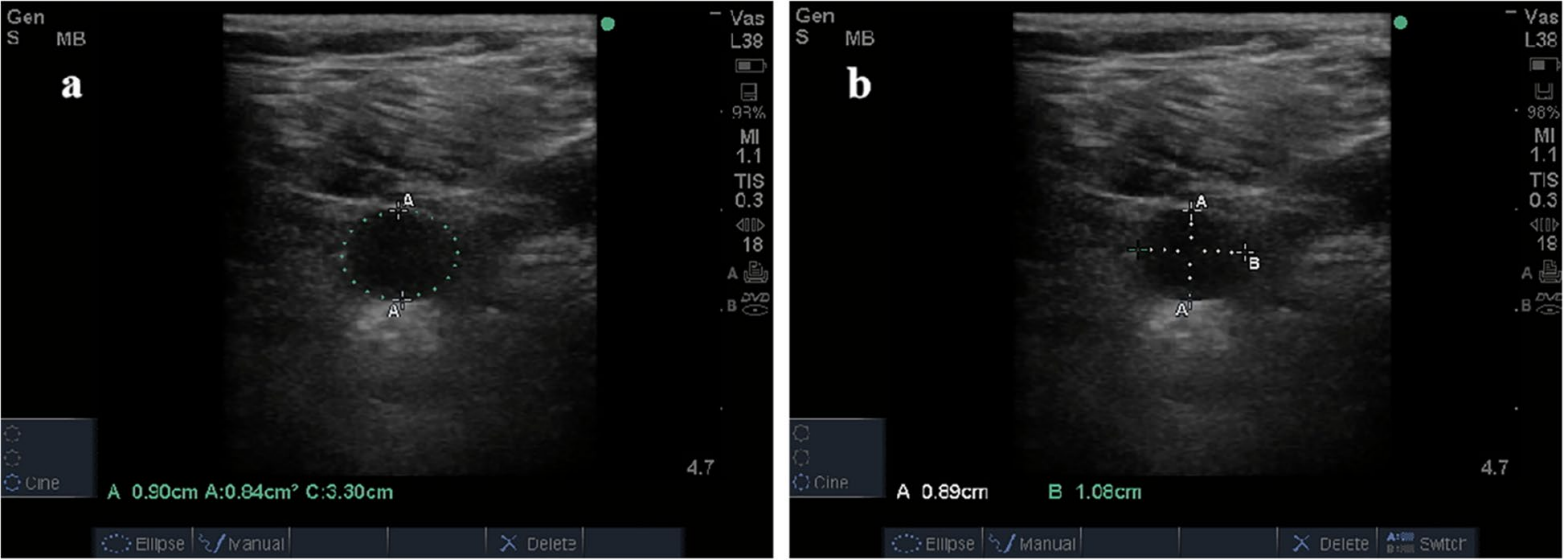
**a** The venous cross-sectional area. **b** Line A is the venous lateromedial diameter and line B is the venous anteroposterior diameter

**Fig. 4:**
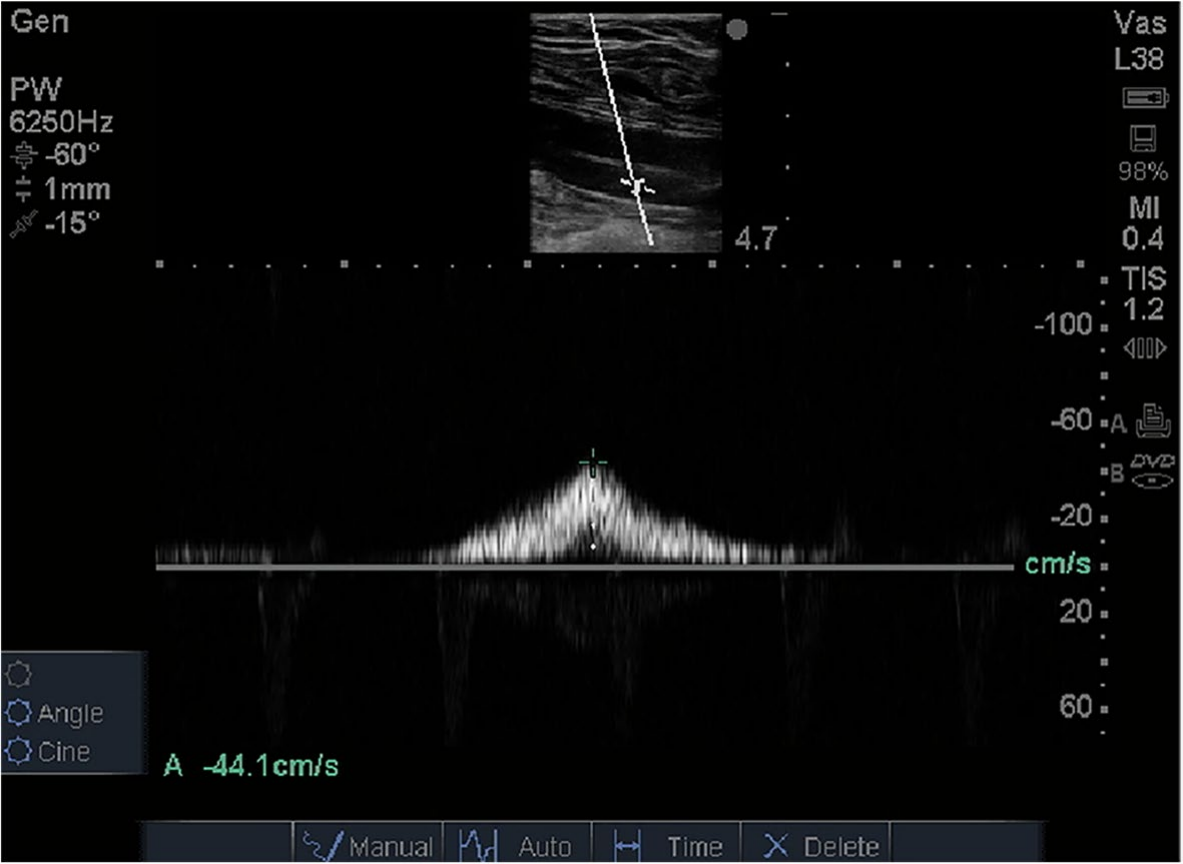
The Doppler cursor was placed in the centre of the vein and peak blood velocity were measured

Ultrasonic Doppler images in the veins were made using a Sonosite M-Turbo ultrasound system. After the scans were completed, the two-dimensional images were automatically generated for venous diameter and cross-sectional area analysis (Fig. 3). Longitudinal scans were performed in the superficial femoral vein. Its peak velocity was measured, and the final value was averaged from the three measurements (Fig. 4).

### Statistical analysis

Data were analysed using SPSS v. 26 (IBM Corp., Armonk, NY, USA). And graphs were plotted with GraphPad Prism 9.0 software (GraphPad, San Diego, CA, USA). All data was given as means with 95% confidence intervals (CI), and the statistical differences between means were assessed with the nonparametric Wilcoxon test. *P* values less than 0.05 (*P* < 0.05) were regarded as statistically significant.

## Results

In total, sixteen patients, who underwent knee osteoarthritis (10 patients), hip osteoarthritis (2 patients), femoral head necrosis (2 patients) and congenital hip dysplasia (2 patients), were included in this study.

### The calf-vein deformation before and after wearing GCS

**Fig. 5:**
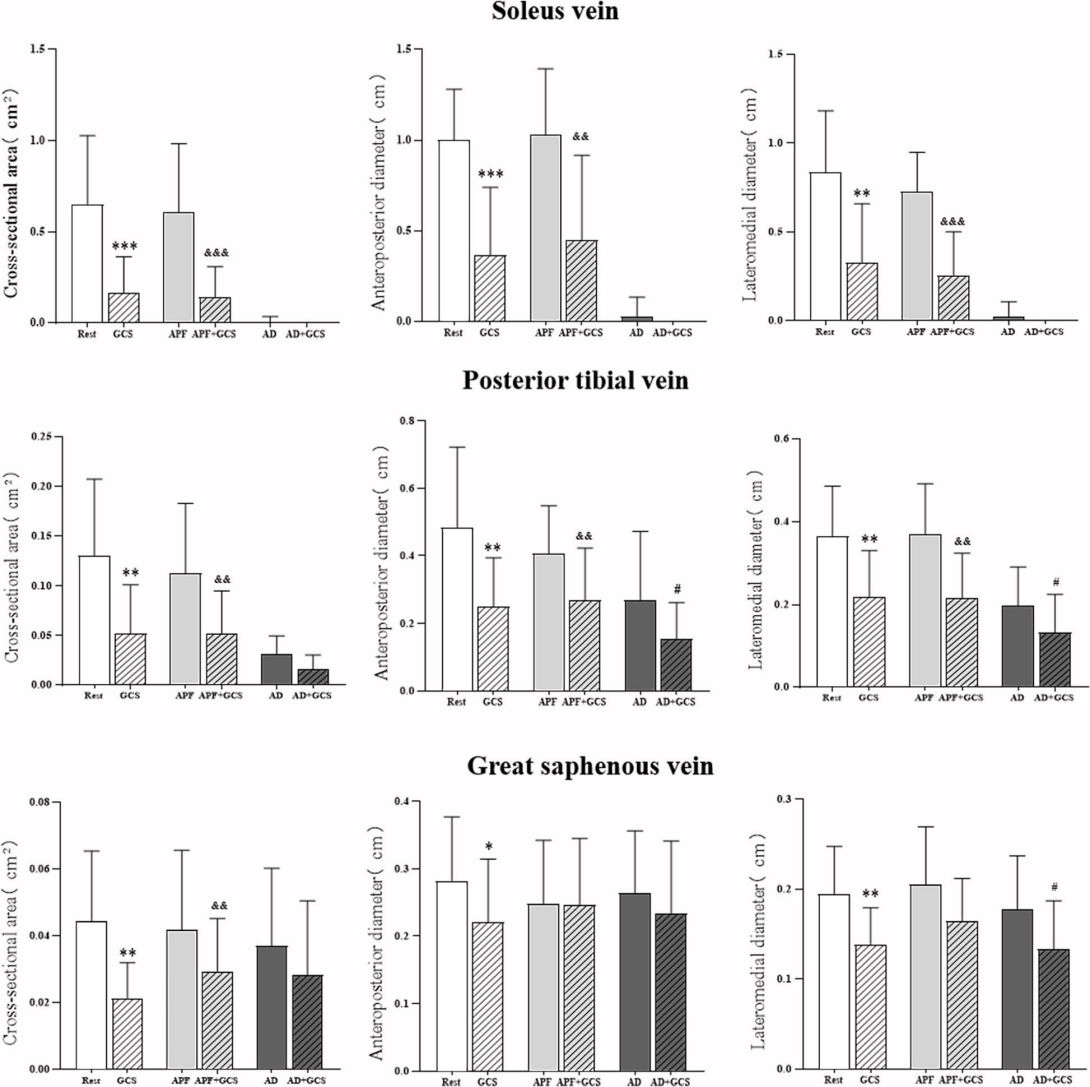
Cross-sectional area, anteroposterior diameter and lateromedial diameter of the soleus vein, posterior tibial vein, and great saphenous vein in the different states (Rest, GCS alone (GCS), Ankle plantar flexion (APF), combined GCS and Ankle plantar flexion (GCS + APF), Ankle dorsiflexion (AD), and combined GCS and Ankle dorsiflexion (GCS + AD)). Error bars indicate SD. Values significantly different from Rest: ^*^p < 0.05; ^**^p < 0.01; ^***^p < 0.001. Values significantly different from Ankle plantar flexion: ^&^*p* < 0.05; ^&&^*p* < 0.01; ^&&&^*p* < 0.001. Values significantly different from Ankle dorsiflexion: ^#^*p* < 0.05; ^##^*p* < 0.01; ^###^*p* < 0.001

Cross-sectional area values for three veins measured in the present study are shown in Table 1 and Fig. 5. With GCS while supine at rest, the cross-sectional area of the SV decreased from 0.65 cm^2^ (95% CI = 0.45–0.85 cm^2^) to 0.17 cm^2^ (95% CI = 0.06–0.27 cm^2^) (*P* < 0.001). The cross-sectional area of the PTV similarly differed in a GCS-related manner (0.13 cm^2^ (95% CI = 0.09–0.17 cm^2^) vs. 0.05 cm^2^ (95% CI = 0.03–0.08 cm^2^); *P* < 0.01), and the GSV cross-sectional area declined from 0.04 cm^2^ (95% CI = 0.03–0.06 cm^2^) to 0.02 cm^2^ (95% CI = 0.02–0.03 cm^2^) (*P* < 0.01) when patients wore GCS.

**Table 1:** The calf-vein deformation in compressed states

	Rest	GCS	APF	APF + GCS	AD	AD + GCS
SV CSA (cm^2^)	0.65 (0.45–0.85)	0.17 (0.06–0.27)	0.61 (0.41–0.81)	0.14 (0.05–0.23)	0.01 (-0.01–0.02)	0 (0–0)
SV diameter ap (cm)	1.00 (0.85–1.15)	0.36 (0.16–0.57)	1.03 (0.84–1.22)	0.45 (0.20–0.70)	0.27 (-0.03–0.08)	0 (0–0)
SV diameter lm (cm)	0.84 (0.65–1.02)	0.33 (0.15–0.50)	0.73 (0.61–0.84)	0.26 (0.13–0.39)	0.02 (-0.02–0.07)	0 (0–0)
PTV CSA (cm^2^)	0.13 (0.09–0.17)	0.05 (0.03–0.08)	0.11 (0.08–0.15)	0.05 (0.03–0.07)	0.03 (0.02–0.04)	0.02 (0.01–0.03)
PTV diameter ap (cm)	0.48 (0.36–0.61)	0.25 (0.18–0.33)	0.41 (0.33–0.48)	0.27 (0.19–0.35)	0.27 (0.16–0.38)	0.15 (0.10–0.21)
PTV diameter lm (cm)	0.36 (0.30–0.43)	0.22 (0.16–0.28)	0.37 (0.31–0.44)	0.22 (0.16–0.27)	0.20 (0.15–0.25)	0.13 (0.08–0.18)
GSV CSA (cm^2^	0.04 (0.03–0.06)	0.02 (0.02–0.03)	0.04 (0.03–0.05)	0.03 (0.02–0.04)	0.04 (0.02–0.05)	0.03 (0.02–0.04)
GSV diameter ap (cm)	0.28 (0.23–0.33)	0.22 (0.17–0.27)	0.25 (0.20–0.30)	0.25 (0.19–0.30)	0.26 (0.21–0.31)	0.23 (0.18–0.29)
GSV diameter lm (cm)	0.19 (0.17–0.22)	0.14 (0.12–0.16)	0.21 (0.17–0.34)	0.16 (0.14–0.19)	0.18 (0.15–0.21)	0.13 (0.11–0.16)

Data are means (95% CI)

*SV* soleus vein, *PTV* posterior tibial vein, *GSV* great saphenous vein

*CSA* cross sectional area, *AP* anteroposterior, *LM* lateromedial

*APF* Ankle plantar flexion, *AD* Ankle dorsiflexion

With maximum ankle plantar flexion, a significant GCS-related reduction in the cross-sectional area of the SV was observed in these patients from 0.61 cm^2^ (95% CI = 0.41–0.81 cm^2^) to 0.14 cm^2^ (95% CI = 0.05–0.23 cm^2^) (*P* < 0.001). Similarly, GCS application reduced the cross-sectional area of the PTV from 0.11 cm^2^ (95% CI = 0.08–0.15 cm^2^) to 0.05 cm^2^ (95% CI = 0.03–0.07 cm^2^) (*P* < 0.01), and the cross-sectional area of the GSV decreased from 0.04 cm^2^ (95% CI = 0.03–0.05 cm2) to 0.03 cm^2^ (95% CI = 0.02–0.04 cm^2^) (*P* < 0.01) when patients wore GCS during the ankle plantar flexion.

With maximum ankle dorsiflexion, GCS application reduced the cross-sectional area of the PTV from 0.03 cm^2^ (95% CI = 0.02–0.04 cm^2^) to 0.02 cm^2^ (95% CI = 0.01–0.03 cm^2^) (*P* < 0.01). However, no significant difference in the cross-sectional area of the SV and GSV were shown with stockings compared to no stockings.

### Femoral vein peak flow velocity

**Fig. 6:**
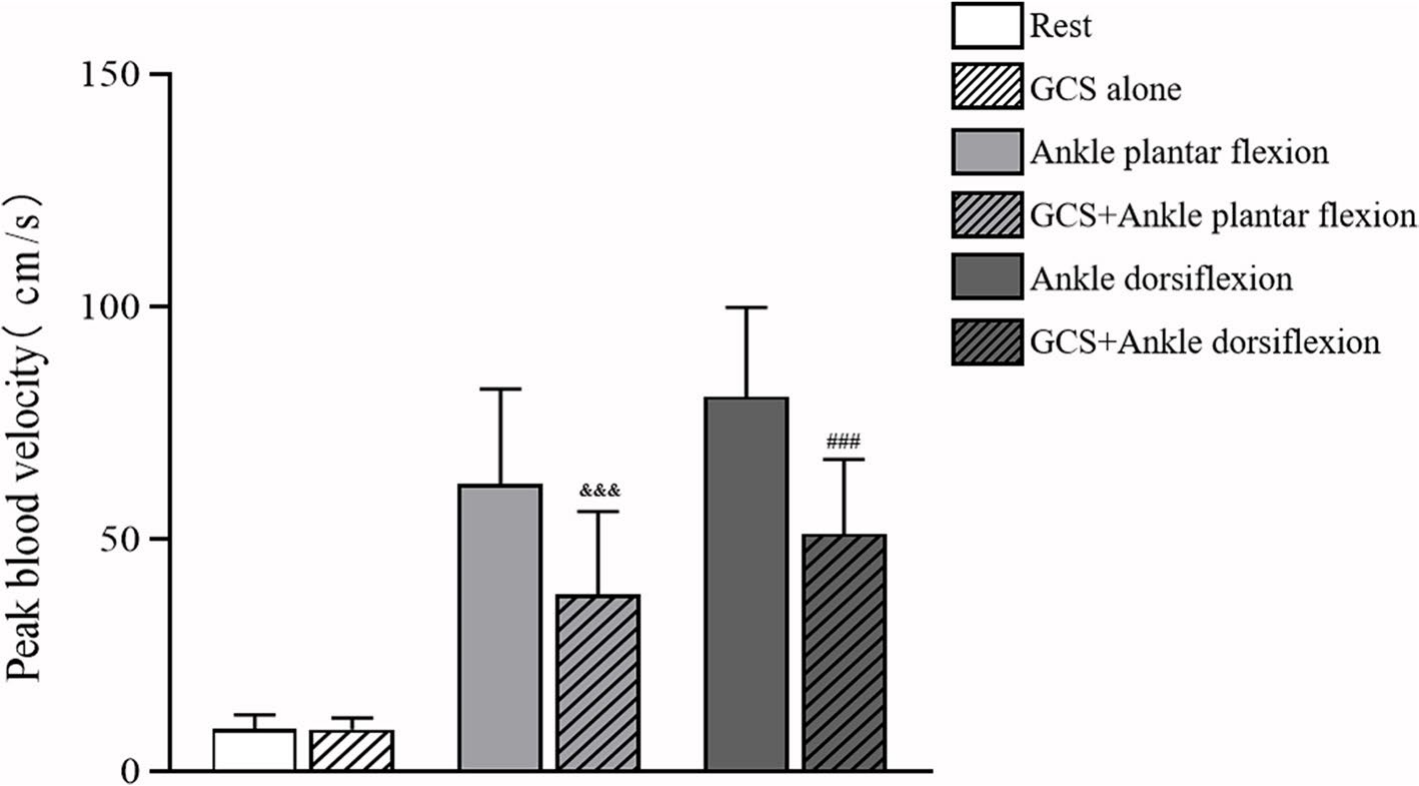
Femoral venous peak velocity in the different states (Rest, GCS alone, Ankle plantar flexion, combined GCS and Ankle plantar flexion, Ankle dorsiflexion, and combined GCS and Ankle dorsiflexion). Error bars indicate SD. Values significantly different from Rest: ^*^p < 0.05; ^**^p < 0.01; ^***^p < 0.001. Values significantly different from Ankle plantar flexion: ^&^*p* < 0.05; ^&&^*p* < 0.01; ^&&&^*p* < 0.001. Values significantly different from Ankle dorsiflexion: ^#^*p* < 0.05; ^##^*p* < 0.01; ^###^*p* < 0.001

**Fig. 7:**
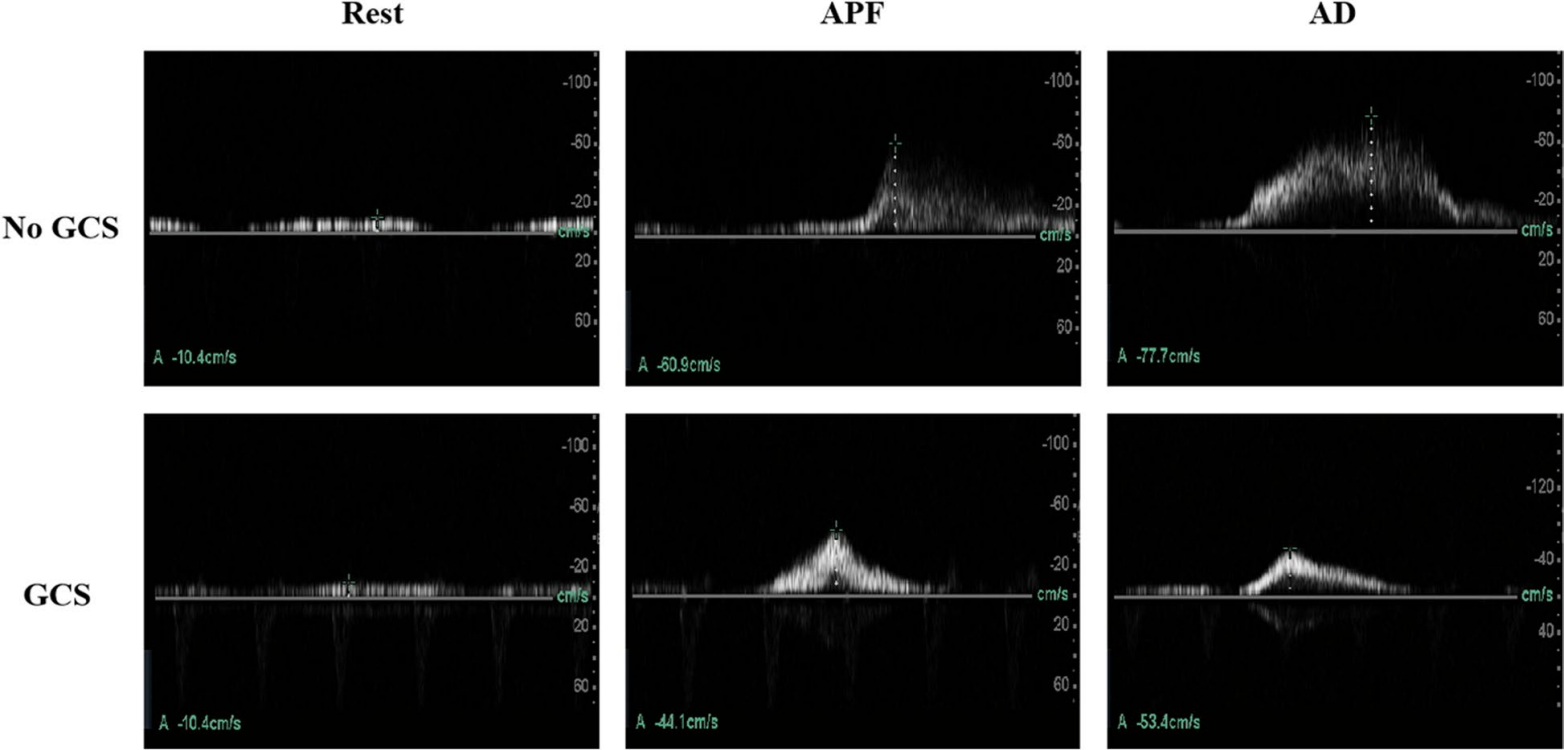
Ultrasonic Doppler images of femoral venous peak velocity during rest, ankle plantar flexion and ankle dorsiflexion, with and without compression

With GCS, the femoral vein peak flow velocity did not differ significantly, 9.09 cm/s (95% CI = 7.80–10.39 cm/s) compared to the velocity while supine at rest without GCS, the value being 9.22 cm/s (95% CI = 7.66–10.79 cm/s) (Table 2, Fig. 6 and Fig. 7).

**Table 2:** The femoral venous peak velocity with and without GCS

	Rest	APF	AD
No stocking	9.22 (7.66–10.79)	61.85 (50.94–72.75)	80.65 (70.37–90.92)
Stocking	9.09 (7.80–10.39)	38.01 (28.42–47.59)	51.15 (42.58–59.73)

Data are means (95% CI)

*APF* Ankle plantar flexion, *AD* Ankle dorsiflexion

During ankle plantar flexion, GCS application reduced the peak flow velocity of the femoral vein in these patients from 61.85 cm/s (95% CI = 50.94–72.75 cm/s) to 38.01 cm/s (95% CI = 28.42–47.59 cm/s) (*P* < 0.001).

During ankle dorsiflexion, a significant GCS-related reduction in the peak flow velocity of the femoral vein was observed in these patients from 80.65 cm/s (95% CI = 70.37–90.92 cm/s) to 51.15 cm/s (95% CI = 42.58–59.73 cm/s) (*P* < 0.001).

## Discussion

This study was designed to explore the effect of GCS on lower extremity venous blood flow velocity and venous deformation during ankle pump exercise. In our study, we found that GCS could significantly decrease the cross-sectional area of SV, PTV and GSV in supine position at rest and maximum ankle plantar flexion. But the compression effect of GCS to SV and GSV was not observed during maximum ankle dorsiflexion. We also found that GCS application significantly reduced the peak flow velocity of the femoral vein during ankle plantar flexion and ankle dorsiflexion, but this effect was not significant in supine position at rest.

These results of the calf-vein deformation agreed with our previous study, which have indicated that knee-length GCS can significantly compress most measured veins including the SV, gastrocnemius vein (GV), PTV, fibular vein, anterior tibial vein and GSV while at rest in the supine position in elderly patients awaiting total knee arthroplasty [21]. Similarly, Partsch et al. [28] determined that low pressure compression stocking can reduce the cross section of superficial and deep veins while in the supine position. Another study conducted by Jeanneret et al. [29] also found that measured parameters including the lateromedial diameter and anteroposterior diameter of the calf veins (GV, PTV and short saphenous vein) were significantly smaller in the prone position when wearing GCS. However, there are also some different results. In the standing position, Jeanneret et al. [29] and Rastel et al. [30] found that the results may not be the same, the vein diameter of the SV, PTV and fibular vein would not be compressed by compression stockings. The cross-sectional area changes of the SV and GSV before and after wearing GCS during maximum ankle dorsiflexion were not in accordance with our expected results. With maximum ankle dorsiflexion, soleus veins were closed in 15 of these 16 patients and were closed in all of the 16 patients in the context of GCS-mediated compression. So, we were unable to determine whether there was a statistical difference before and after wearing GCS. The cross-sectional area changes of the GSV after wearing GCS during maximum ankle dorsiflexion, eight patients reported an increase, and four patients reported no change, four patients reported a decrease. The cross-sectional area of GSV is very small, so there may be errors in the measurement. And a significant GCS-related reduction in the lateromedial diameter of the GSV was observed in these patients with maximum ankle dorsiflexion. Consequently, we considered that GCS can significantly compress GSV during maximum ankle dorsiflexion.

Previous research has mainly focused on observing the changes in lower limb venous flow velocities during ankle pump exercise whereas research about the calf-vein deformation is sparse [24, 25, 31]. In this study, we focused on the calf-vein deformation in three positions of the leg (flat, maximum ankle plantar flexion and maximum ankle dorsiflexion). And we show for the first time that soleus veins were completely closed in 15 of these 16 patients with maximum ankle dorsiflexion. The possible reason for this phenomenon is that the gastrocnemius muscle and soleus muscle of the patients was lengthened during maximum ankle dorsiflexion. And the veins are therefore subjected to a more uniform pressure similar to intermuscular pressure. Some studies found that the intermuscular vein is the location where DVT often occurs, and soleus vein dilatation is an independent risk factor for deep venous thrombosis after orthopedic surgery [32, 33]. Thus, the results obtained in this study could be considered as one of the possible mechanisms to explain that ankle pump exercise can prevent intramuscular venous thrombosis, which also needs to be further studied.

To our surprise, our results were entirely counter to our hypothesis. This study showed that GCS application significantly reduced the peak flow velocity of the femoral vein during ankle exercise. This outcome is also contrary to that of Stein et al. [34] who found that thigh-length GCS did produce a further increase in the time-averaged peak velocity of the popliteal vein during supine ankle exercise. Espeit et al. [35] concluded that the combination of GCS and local vibration could increase the peak velocity of the popliteal vein than local vibration alone. And Sakai et al. [27] showed that intermittent pneumatic compression with active ankle exercise lead to a significant increase in femoral vein peak venous velocity compared to active ankle exercise alone. There are also some prior studies that were consistent with our results. Warwick et al. [36] found that GCS application significantly reduced the popliteal venous peak velocity in each position of the leg (foot-up, flat and foot-down) when the AV Impulse Foot Pump was activated. This result may be explained by the fact that a reduced preload of the foot venous plexus would limit the volume available for expulsion.

As we know, ankle pump exercise can promote lower extremity venous reflux by contracting and relaxing gastrocnemius and soleus muscles. In this study, it was observed that GCS application reduced the cross-sectional area of the calf veins, which might restrict the preload of the veins. And this restriction in its preload induced a reduction in the volume available for expulsion, which eventually caused a decrease in the peak flow velocity of the femoral vein when GCS was used simultaneously with ankle pump exercise. Another possible reason is that muscle contraction and relaxation are time limited. So, if the contraction and relaxation of muscle is too fast, the pumping and reflux of blood will be affected.

Peak flow velocity of the lower extremity is considered as an alternative measure for preventing thrombosis [37]. And a comparative prospective trial involving 800 patients also showed that the incidence of thromboembolism after total hip or knee replacement did not differ between patients treated with foot pumps alone and with the combination of foot pumps and GCS. Nevertheless, foot pumps without GCS could also increase patient compliance [38]. Thus, what we need to do is optimizing the use of GCS in combination with ankle pump exercise in the patients after joint replacement surgery, from these standpoints. To best improve venous hemodynamic of patients after joint replacement, it is critical to find more suitable frequency, intensity, duration and so on during ankle pump exercise. The specific practices need to be further studied.

This study has some limitations. Firstly, in the present study, the movements of the ankle joint focused solely on the dorsiflexion and plantarflexion. Other movements consisted of eversion, inversion, internal rotation and external rotation might lead to different outcomes. Furthermore, we hypothesized that the results in the patients before joint replacement could be applied to patients after joint replacement. Although studies involving postoperative patients would be more relevant to confirm our outcomes, the accuracy of the results may be affected due to the patient’s post-operative pain, discomfort and swelling. Thirdly, we focused exclusively on knee-length GCS and did not assess the possible effects of thigh-length GCS. Some studies found that thigh-length GCS enables better improvement of venous hemodynamic [34, 39]. Finally, there are no randomized clinical trials to confirm the question if the stockings should be used with ankle pump exercise.

## Conclusion

In conclusion, the results of this study showed that GCS application significantly reduced the peak flow velocity of the femoral vein during ankle exercise in the patients before joint replacement. We suggest further optimization of the use of GCS in combination with rehabilitation exercise. And there should be more focus on this problem when using GCS for the prevention of DVT in clinical work.

## Data Availability

All datasets used and analyzed during the current study are available from the corresponding author on reasonable request.

## Abbreviations

GCS: Graduated compression stockings
CSA: Cross-sectional area
AP: Anteroposterior
LM: Lateromedial
SV: Soleus vein
PTV: Posterior tibial vein
GSV: Great saphenous vein
FV: Femora vein
DVT: Deep vein thrombosis
CI: Confidence intervals
APF: Ankle plantar flexion
AD: Ankle dorsiflexion
GV: Gastrocnemius vein
